# Balloon Pump with Floating Valves for Portable Liquid Delivery

**DOI:** 10.3390/mi7030039

**Published:** 2016-03-01

**Authors:** Yuya Morimoto, Yumi Mukouyama, Shohei Habasaki, Shoji Takeuchi

**Affiliations:** 1 Center for International Research on Integrative Biomedical Systems (CIBiS), Institute of Industrial Science (IIS), The University of Tokyo, 4-6-1 Komaba, Meguro-ku, Tokyo 153-8505, Japan; y-morimo@iis.u-tokyo.ac.jp (Y.M.); yumi.mukouyama@gmail.com (Y.M.); habasaki@iis.u-tokyo.ac.jp (S.H.); 2 Takeuchi biohybrid Innovation Project, Exploratory Research for Advanced Technology (ERATO), Japan Science and Technology (JST), Komaba Open Laboratory (KOL) Room M202, 4-6-1, Komaba, Meguro-ku, Tokyo 153-8904, Japan

**Keywords:** microfluidic device, portable device, optofluidic lithography

## Abstract

In this paper, we propose a balloon pump with floating valves to control the discharge flow rates of sample solutions. Because the floating valves were made from a photoreactive resin, the shapes of the floating valves could be controlled by employing different exposure patterns without any change in the pump configurations. Owing to the simple preparation process of the pump, we succeeded in changing the discharge flow rates in accordance with the number and length of the floating valves. Because our methods could be used to easily prepare balloon pumps with arbitrary discharge properties, we achieved several microfluidic operations by the integration of the balloon pumps with microfluidic devices. Therefore, we believe that the balloon pump with floating valves will be a useful driving component for portable microfluidic systems.

## 1. Introduction

Microfluidic devices have been extensively developed to enable their application in chemical, biological, and biomedical processes because of their rapid processing and high sensitivity by minimizing the sample size [1,2]. Recently, integrated systems comprising microfluidic devices and liquid feed devices have garnered attention for use in point-of-care diagnostic testing and scientific studies without employing expensive and bulky equipment [3,4]. In such systems, the liquid feed devices with driving sources enable the precise and continuous control of samples [5,6,7,8,9,10]; however, the use of these devices result in low application possibilities for *in situ* use of the systems because the driving sources have low portability and need external components (e.g., electric sources) for their operation.

To solve the abovementioned problem, portable microfluidic systems based on osmotic pressure [11,12], capillary flow [13], negative pressure generated using a vacuumed chamber [14,15], surface tension of droplets [16], and finger power [17,18,19,20] have been developed to generate flow in microfluidic channels without the use of external components. Although these microfluidic systems allow *in situ* use as portable devices, they do not provide continuous liquid delivery and closed channels and/or inlets for preventing the evaporation and contamination of liquids. As an improved microfluidic system that can solve the abovementioned issues, Gong *et al.* integrated a balloon pump with a syringe to manipulate samples in a closed system [21]. When samples are infused via the syringe, the balloon pump allows the storage of liquids inside it by inflating the balloon and facilitates the transport of liquids by deflating the balloon. Therefore, the balloon pump provides continuous liquid delivery and ensures the conservation of independent environments, thereby making it an attractive candidate for use as a portable microfluidic system. However, the discharge liquid flow rates cannot be easily regulated in the balloon pump because the flow rates depend on the deflation pressure of the balloon. To change the flow rates, the balloon pump needs to be redesigned to incorporate additional components such as microchannels that work as flow resistors using specialized equipment for microfabrication.

**Figure 1:**
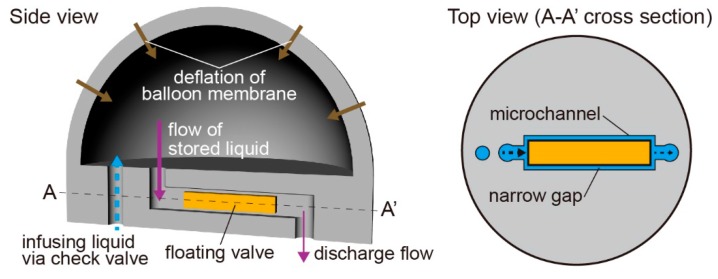
Conceptual illustration of a balloon pump with a floating valve.

In this study, we develop a balloon pump with floating valves to change the flow rates of liquids (Figure 1). The advantages of the balloon pumps are: (i) fabrication of floating valves without specialized equipment for microfabrication; (ii) changeable discharge flow properties by conditions of the floating valves; and (iii) liquid delivery without any additional manipulation. As a fabrication technique for floating valves, we apply optofluidic lithography to a photoreactive resin in microfluidic channels made from polydimethylsiloxane (PDMS) [22]. By mounting an exposure system to a microscope, we can prepare the floating valves in the balloon pump without use of any microfabrication techniques. Using this method, we can control the length and width of the floating valves by changing the exposure pattern. Furthermore, we can vary the shapes of the floating valves in the PDMS microchannels by changing the oxygen concentration in the balloon pump because the oxygen absorbed in the PDMS channels inhibits the polymerization of the photoreactive resin [23]. By controlling the floating valve dimensions, we can easily prepare flow resistors with arbitrary properties and hence control the properties of liquid discharge. The floating valves are placed in the microchannels and create narrow gaps with the walls of the microchannels. Because the narrow gaps can act as flow resistors and decrease the flow rates [24,25], the balloon pump can change the discharge flow rates according to the dimensions of the floating valves. Consequently, as a demonstration of microfluidic operations using the proposed balloon pump, we present the manipulation of microsized beads in dynamic microarray devices [26] and the formation of laminar flows. Because these microfluidic operations need flow control, the demonstration indicates the potential of the balloon pumps for portable microfluidic systems to easily generate controlled flows without the use of external driving elements.

## 2. Experimental

### 2.1. Materials

For the fabrication of the balloon pump, we used PDMS and a curing agent (Sylgard 184 Silicone Elastomer, Dow Corning Toray Co., Ltd., Tokyo, Japan), a photoreactive acrylate resin (R11, 25–50 μm layers, EnvisionTEC, Dearborn, MI, USA), parylene (parylene-C, Specialty Coating Systems, Inc., Indianapolis, IN, USA), and SU-8 (SU-8 50, MicroChem Corp., Westborough, MA, USA). The materials used for manufacturing the floating valves were polyethylene glycol diacrylate (PEGDA) (Sigma-Aldrich, St. Louis, MO, USA, average *M*n = 700) and phenylbis ((2,4,6-trimethylbenzoyl)phosphine oxide) (Sigma-Aldrich). To demonstrate the microfluidic operations of the balloon pump with the floating valves, we used 100 μm beads (PS-Red-Particles, microParticles GmbH, Berlin, Germany), Tween 20 (Kanto Chemical Co., Inc., Tokyo, Japan), and blue ink (Pilot Corp., Tokyo, Japan).

### 2.2. Device Design and Fabrication

Figure 1 shows a schematic illustration of the balloon pump with the floating valves. The balloon pump works as a pneumatically driven pump because of the inflation and deflation of the balloon membrane. In addition, the floating valves work as flow resistors by the formation of narrow flow paths with the channel walls. By adjusting the width and length of the flow paths, we can control the discharge characteristics of the balloon pump.

To fabricate the balloon pump, we integrated a balloon layer, an intermediate layer, and a microchannel layer, all made from PDMS. We shaped these three layers by molding. The molds for the balloon layer and intermediate layer were made by carrying out stereolighography on a photoreactive acrylate resin using a modeling machine (Perfactory, EnvisionTEC). After the fabrication of the molds, we exposed them to ultraviolet (UV) light for over 60 s using a laser machine (UV-LED, Keyence Corp., Osaka, Japan) to ensure complete curing. Then, we coated them with a 2 μm parylene layer using a chemical vapor deposition machine (Parylene Deposition System 2010, Specialty Coating Systems, Inc.) to avoid direct contacts between PDMS and the surface of the resin mold that cause non-solidification of PDMS. The mold for the microchannel layer was made from SU-8 by standard soft lithography techniques. After spin-coating SU-8 on a silicon wafer to form a layer with a height of 60 μm, we fabricated the SU-8 mold by UV light exposure through a photomask (Clean Surface Technology Co., Ltd., Kanagawa, Japan) designed using a mask exposure machine (D-Light DL-1000, NanoSystem Solutions, Inc., Okinawa, Japan).

**Figure 2:**
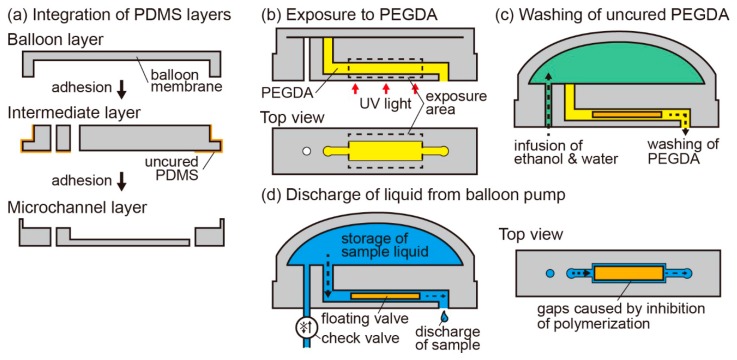
Process flow of the preparation of a balloon pump with a floating valve: (**a**) fabrication of the balloon pump by the integration of polydimethylsiloxane (PDMS) layers; (**b**) solidification of polyethylene glycol diacrylate (PEGDA) using UV light in a microchannel; (**c**) washing of uncured PEGDA using ethanol and water; and (**d**) discharge of the liquid from the balloon pump after the liquid is infused via a check valve.

We filled the three molds with a PDMS-curing agent mixture in the ratio of 10:1 (*w*/*w*). After peeling the cured PDMS layers from the molds manually without using any organic solvents, we coated a specific surface of the intermediate layer with pre-cured PDMS using a pipette and heated it for 55 min at 60 °C to fabricate a semi-cured PDMS layer. Finally, we bonded all layers by heating them again for 90 min at 75 °C (Figure 2a).

Next, we constructed the floating valves in the microchannel of the balloon pump. First, we prepared PEGDA with 1% (*w*/*v*) of phenylbis as the photoreactive resin for the floating valves. The photoreactive resin was introduced into the microchannel via an outlet while ensuring that the resin did not touch the balloon membrane. Then, we exposed the resin to UV light in the required shapes of the valves using a microscope (IX71, Olympus Corp., Tokyo, Japan) equipped with a digital micromirror device (DMD) (Figure 2b). Using the DMD, we were able to make the floating valves in arbitrary two-dimensional extruded shapes by changing the exposure patterns, and we were able to fabricate multiple floating valves in one microchannel by changing the exposure area. After the exposure process, we washed the microchannel by infusing ethanol and water via an inlet and aspirating them from an outlet to remove the non-cured resin (Figure 2c). The fabricated valves did not stick to the wall of the microchannel because the oxygen layers on the PDMS surface worked as a PEGDA polymerization inhibitor; therefore, liquids could pass though the gaps between the microchannel wall and floating valve. In the fabrication process of the floating valves, the length of the valve in the flow direction was longer than that of the exposure pattern because of the flow of the photoreactive resin in the microchannel. To make up for the difference between the two lengths, we adjusted the length of the exposure pattern according to a calibration line (Supplementary Figure S1). To increase the widths of the floating valves, we degassed the PDMS devices in a vacuum desiccator (AS ONE Corp., Osaka, Japan), to reduce the thickness of the oxygen layers.

### 2.3. Setup of the Balloon Pump

We attached a check valve (PU Celsite Port, Toray Medical Co., Ltd., Tokyo, Japan) at the inlet of the balloon pump to prevent backward flow of liquids. Then, we carefully infused liquids into the balloon pump via the check valve using a syringe to avoid trapping air bubbles under the balloon membrane. In case air bubbles were trapped, we infused water again after removal of the previously loaded water with air bubbles. Because of the actions of the check valve and floating valve, the balloon pump stored liquids by the inflation of the balloon membrane. Because liquids could pass through the gaps between the floating valves and channel walls, the pump gradually discharged the liquids by the deflation of the balloon (Figure 2d).

### 2.4. Evaluation of the Balloon Pump

To evaluate the behavior of the balloon pumps, we checked their external appearance and their discharge characteristics. The external appearance of the balloon pumps was obtained by taking pictures using a digital camera (EOS Kiss X6i, Canon Inc., Tokyo, Japan). For checking the discharge characteristics of the balloon pumps, we stored water in the pump using a syringe and check valve and measured the volume of the discharge water at the outlet of the balloon pump using a pipette.

To check the flow regulation properties of the floating valve in the microchannels, we prepared floating valves in the PDMS microchannels composed of the intermediate layer and microchannel layer; this system was used to control the input flow pressure using a pressure-driven flow pump (MFCS-100, Fluigent, Inc., Villejuif, France). In the experiment, we measured the volume of the discharge water at the outlet of the microchannel using a pipette.

For evaluating the characteristics of the discharge caused by the deflation of the balloon membrane, we prepared devices composed of the balloon layer and intermediate layer to eliminate the effects of the microchannel. We controlled the volume of the stored water using a syringe pump (KDS210, KD Scientific, Holliston, MA, USA) and connected a pressure gauge (GP-M 001, Keyence Corp.) to an outlet of the device to measure the output pressure of the discharge water.

### 2.5. Microfluidic Operations Using the Balloon Pump

We integrated laminar flow devices and dynamic microarray devices with the outlet of the balloon pump. Both devices were prepared by bonding PDMS channels and glass plates. In the case of the demonstration using laminar flow devices, we connected the balloon pumps containing water and water including 10% (*v*/*v*) blue ink separately to two inlets of the laminar flow device. The dynamic microarray devices enabled us to make arrays of microsized beads. For the demonstration using the dynamic microarray devices, we placed microsized beads at an inlet of the dynamic microarray device and then connected the balloon pump containing water with 0.5 wt % Tween 20 to the device. The discharge water from the balloon pump pushed the stored microsized beads to the dynamic microarray devices. In both the experiments, we used a microscope (IX71, Olympus Corp.) for observation.

## 3. Results and Discussion

### 3.1. Regulation Properties of the Floating Valve

**Figure 3:**
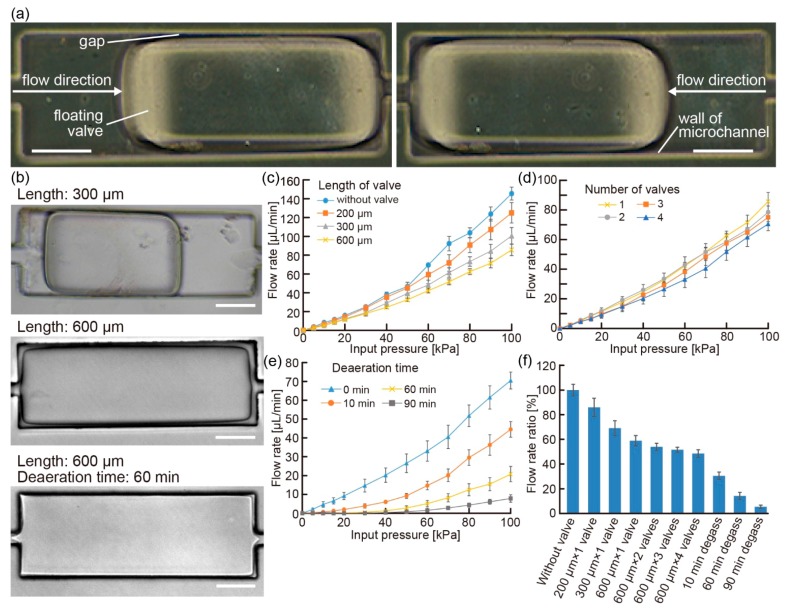
(**a**) Images of the floating valve in motion according to the flow direction (scale bars: 100 μm); (**b**) images of the floating valves with different shapes controlled by the exposure pattern and degassing time (scale bars: 100 μm); (**c**–**e**) changes in the regulation properties of the floating valves with varying input pressure of liquids when the (c) length, (d) number (length: 600 μm), and (e) degassing time of the floating valves were changed; and (**f**) summary of the regulation properties of the floating valves fabricated under different conditions at 100 kPa input pressure.

The floating valves moved freely in the microchannel according to the flow direction since the oxygen layer on the PDMS surface worked as a PEGDA polymerization inhibitor (Figure 3a) (Movie S1). In this state, by designing an appropriate exposure pattern using the DMD, we succeeded in controlling the shape of the floating valve (Figure 3b). In addition, by shrinking the oxygen layer on the PDMS channel surface using a degassed balloon pump, larger floating valves were fabricated in the channels (Figure 3b). These results show that our fabrication method can change the configuration of the balloon pump easily without any change in the fabrication tools such as photomasks and molds.

To evaluate the floating valves, we checked their flow regulation properties in the microchannel against the input flow pressure using the pressure-driven pump. Using the floating valves with various shapes, we could change the discharge flow rates under the same input flow pressure by controlling the length and number of the valves (Figure 3c,d): when the number or length of the valves increased, the discharge flow rates decreased. The results indicate that the formation of long gaps between the valves and channel walls can result in small flow rates because an increase in the length and number of the valves causes the gap to be long. We believe that the gaps caused the flow path to become narrow and regulated the flow speed because of a loss in the flow pressure. In addition, these results showed that slopes of discharge flow rates increased according to increase of the input flow pressures differently from expected principle of Hagen-Poiseuille flow. We think that extension of the PDMS microchannels by applied pressures caused accelerated increase of the discharge flow rates because we observed that applied pressure deformed the microchannels (Movie S1). Furthermore, we managed to decrease the discharge flow rates by changing the degassing time of the PDMS devices (Figure 3e). The use of degassed PDMS devices for a longer period (up to 90 min) caused the floating valves to generate flows with lower flow rates since the gaps between the valves and channel walls became narrower. However, degassing for over 120 min caused clogging of the microchannel because of the dissipation of the oxygen layer on the PDMS surface. From these results, we confirmed that the configuration changes of the floating valves allowed us to easily control the discharge characteristics at the same input flow pressure (Figure 3f). Because various microchannels with an extensive range of lengths were necessary to facilitate the control of the discharge characteristics (Figure S2), the floating valves were appropriate to control the flow rates of the balloon pumps without requiring any design changes of the microchannels.

### 3.2. Discharge Characteristics of the Balloon Pump

For the evaluation of the balloon pump, we investigated the inflation and deflation properties of the balloon membranes (diameter: 15 mm). First, we checked the maximum amount of stored water by the inflation of the balloon membrane. We prepared balloon membranes with different thicknesses and infused water using a syringe until leakage started. From the results, we confirmed that the balloon pump with a thinner balloon membrane could store more amount of water than that with a thicker membrane (Figure S3a). On the other hand, when we measured the deflation pressure of the balloon membrane using the pressure gauge, we found that thick balloon membranes were necessary to achieve reproducible deflation of the membrane (Figure S3b). These results show that a balloon membrane with an appropriate thickness is required to satisfy the conditions of a good volume of stored liquids as well as reproducible deflation. Therefore, we used a balloon membrane with 0.4 mm thickness in the balloon pumps for further experiments.

**Figure 4:**
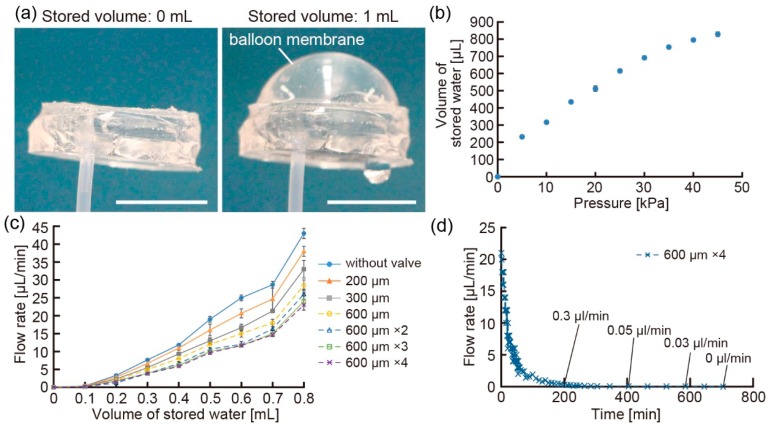
(**a**) Images of the inflation of the balloon membrane when storing water (scale bars: 1 cm); (**b**) relationship between the maximum volume of stored water and the applied input pressure; (**c**,**d**) plots of the discharge flow rates for different floating valves according to the (c) volume of stored water and (d) time after the storage of 0.8 mL of water.

Using the balloon pump, we discharged liquids without using any external driving source such as an electrical source. When we infused water into the balloon pump via the check valve, the balloon pump stored water in the balloon membrane without any leakage (Figure 4a). To analyze the properties of the balloon membrane, we clarified the relationship between the volume of the stored water and the flow pressure applied using a pressure-driven pump (Figure 4b). The results indicate that the input flow pressure induced by the deflation of the balloon membrane changes according to the volume of the stored water. Because of the change in the input flow pressure, the discharge flow rates of the balloon pumps varied in response to the volume of the stored water (Figure 4c). In this state, we analyzed the regulation characteristics of the floating valves and found that when the number and length of the floating valves increased, the discharge flow rates of the balloon pumps decreased. This result shows that the floating valve works as a flow regulator in the balloon pumps. However, we observed that the balloon pumps with the floating valves fabricated in degassed PDMS devices did not deliver water because the input flow pressure of the balloon pumps was not sufficiently more than the loss of pressure around the floating valves. Furthermore, the balloon pump enabled the delivery of water over 10 h, and discharged properties of the balloon pump followed basic balloon principles [21] (Figure 4d). Based on these results, we believe that balloon pumps with floating valves can be used as portable pumps for liquid delivery.

### 3.3. Microfluidic Operations Using the Balloon Pump

**Figure 5:**
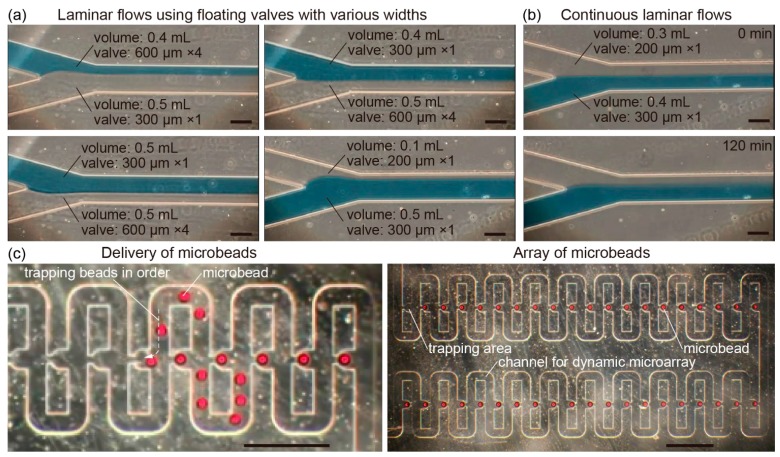
(**a**) Laminar flows formed by using balloon pumps with various floating valves and volumes of stored liquids; (**b**) continuous formation of the laminar flows; and (**c**) formation of an array of microbeads in the dynamic microarray device using the balloon pump with four 600 μm width floating valves and 0.3 mL of stored water. Scale bar: (a,b) 100 μm; and (c) 500 μm.

As a demonstration of the microfluidic operations using the balloon pump, we integrated a laminar flow device and dynamic microarray device with the balloon pumps. We succeeded in forming laminar flows in the device by the liquid delivery of individual balloon pumps. In this state, the balloon pumps can facilitate changes in the widths of the laminar flows by altering the combination of the balloon pumps because we can control the discharge flow rates from the balloon pumps according to the type of the floating valves and the volume of the stored liquids (Figure 5a). Because the balloon pumps provide liquid delivery for more than a few hours, they could also achieve the formation of laminar flows for over 2 h (Figure 5b). In this regard, role of diffusion increased after 2 h because flow rates are substantially smaller due to basic balloon principles. Because some microfluidic devices need the formation of laminar flows for several tens of minutes to prepare fiber-shaped samples [27], the balloon pumps can be used in seamless *in situ* sample fabrication.

Furthermore, when microbeads were placed at the inlet of the dynamic microarray device, the discharge water from the balloon pump delivered the microbeads to the device channels. As a result, we achieved the induction of the microbeads into the trapping area in order and the formation of an array of microbeads (Figure 5c). In addition, the speeds of microbead delivery in the channel were controlled by the type of the floating valves in the balloon pumps (Movie S2). Because the dynamic microarray device allows the use of various types of beads such as polymer capsules with microbes and collagen beads with cells by controlling the speed of sample delivery [28,29], we infer that the balloon pump is a useful tool for the *in situ* array formation of various bead-shaped samples in the dynamic microarray devices.

## 4. Conclusions

In this study, by combining a balloon membrane, microchannels, and floating valves, we developed balloon pumps for *in situ* liquid delivery without the use of external sources. The advantages of the balloon pumps are as follows: (i) easy preparation of various types of floating valves because of photopolymerization using a DMD system; (ii) changeable discharge flow properties by adjusting the number and width of the floating valves; and (iii) liquid delivery without any additional manipulation. Thus, the balloon pumps can be used for *in situ* microfluidic operations instead of conventional pumps. In addition, the balloon pump provides adjustability of discharge flow properties to users who mount a DMD system to a microscope because the users can change dimensions of the floating valves by exposure condition. Therefore, we believe that the balloon pump with the floating valves will be a useful tool for manipulating liquids and samples in microfluidic devices for point-of-care analyses.

## Supplementary Materials

The following are available online at http://www.mdpi.com/2072-666X/7/3/39/s1, Figure S1: Relationship between the lengths of the exposure area and fabricated floating valves, Figure S2: Plots of the discharge flow rate via microchannels with different lengths at various input pressures, Figure S3: Influence of change of balloon membrane thickness, Movie S1: Motion of the floating valve according to the flow direction. Movie S2: Formation of an array of microbeads in the dynamic microarray device using the balloon pumps under various conditions.
