# Supplementary material for: Balloon Pump with Floating Valves for Portable Liquid Delivery
Source: Micromachines (Basel). 2016 Mar 1;7(3):39. doi: 10.3390/mi7030039 (PMC6189947; doi:10.3390/mi7030039)
Supplement: Supplementary file 1 [file micromachines-07-00039-s001.zip › Supplementary Materials.pdf]

Supplementary Materials: Balloon Pump with Floating Valves for Portable Liquid Delivery

Yuya Morimoto, Yumi Mukouyama, Shohei Habasaki and Shoji Takeuchi *

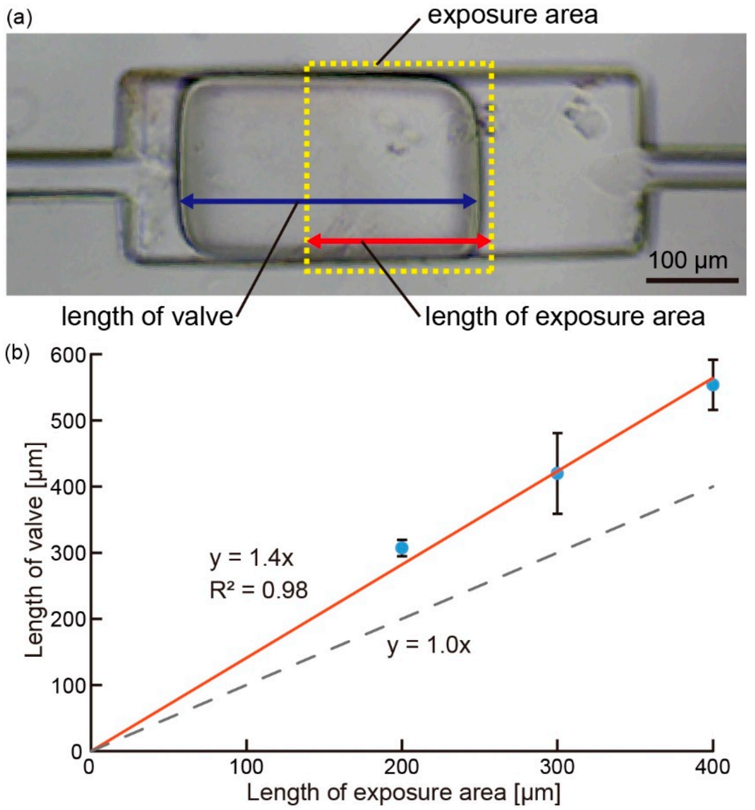

Figure S1. (**a**) Image showing a comparison of the lengths of the exposure area and the floating valve. (**b**) Relationship between the lengths of the exposure area and fabricated valve. The length of the valve was longer than that of the exposure area.

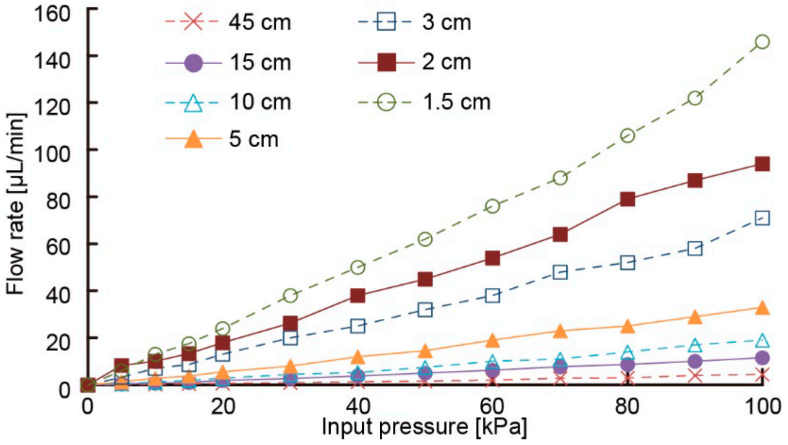

Figure S2. Plots of the discharge flow rate via microchannels (area: 50 μm × 50 μm) with different lengths at various input pressures.

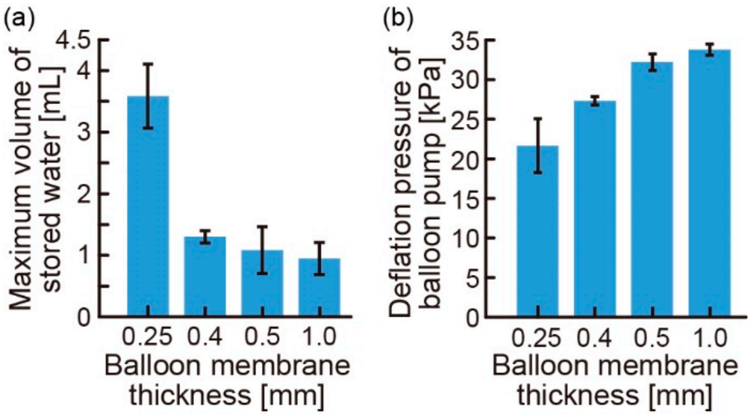

Figure 3S. (**a**) Relationship between the maximum volume of stored water and the thickness of the balloon membrane. (**b**) Relationship between the maximum deflation pressure of the balloon membrane and the thickness of the balloon membrane when 1 mL of water was stored in the pump.
